# A direct comparison of 3 T contrast-enhanced whole-heart coronary cardiovascular magnetic resonance angiography to dual-source computed tomography angiography for detection of coronary artery stenosis: a single-center experience

**DOI:** 10.1186/s12968-020-00630-2

**Published:** 2020-06-01

**Authors:** Bin Sun, Zhiyong Chen, Qing Duan, Yunjing Xue, Lianglong Chen, Zhongshuai Zhang, Jing An

**Affiliations:** 1 Department of Radiology, Union Hospital, Fujian Medical University, 29 Xin-Quan Road, Fuzhou, 350001 People’s Republic of China; 2 Department of Cardiology, Union Hospital, Fujian Medical University, Fuzhou, China; 3 Diagnostic imaging, Siemens Healthcare, Shanghai, China; 4 Siemens Shenzhen Magnetic Resonance Ltd., Shenzhen, China

**Keywords:** Magnetic resonance imaging, Computed tomography, Coronary angiography, Contrast-enhanced

## Abstract

**Background:**

In recent years, substantial advances have been made in noninvasive cardiac imaging, including cardiac computed tomography (CT) and cardiovascular magnetic resonance (CMR). The purpose of this study was to prospectively compare the diagnostic performance of contrast-enhanced whole heart coronary CMR angiography (CCMRA) to dual-source coronary CT angiography (CCTA) for the diagnosis of significant coronary stenoses (≥50%) in patients with known or suspected coronary artery disease (CAD) referred for conventional x-ray coronary angiography.

**Methods:**

Our objective was to directly compare the diagnostic accuracy of contrast-enhanced whole-heart CCMRA (CE-CCMRA) to dual-source CCTA (DS-CCTA) for the detection of CAD. We prospectively studied 57 symptomatic patients with suspected or known CAD who were scheduled for conventional x-ray coronary angiography. Significant CAD was defined as an x-ray defined diameter reduction of ≥50% in a coronary artery with a reference diameter of ≥1.5 mm.

**Results:**

CE-CCMRA and DS-CCTA were completed in 51 (89%) of 57 patients without complications. The acquisition times of CE-CCMRA and DS-CCTA, respectively, were 9.5 ± 3.1 min and 8.3 ± 1.4 s. On patient-based analysis, the sensitivity, specificity, positive and negative predictive value of CE-CCMRA and DS-CCTA were 93.5% versus 93.5%(*P* > 0.05), 85% versus 90%(P > 0.05), 90.6% versus 93.5%(P > 0.05), and 89.4% versus 90%(P > 0.05), respectively. The area under the curve (AUC) was 0.89 (95% CI: 0.79 to 0.99) for CE-CCMRA and 0.92 (95% CI: 0.83 to 1.00) for DS-CCTA.

**Conclusions:**

DS-CCTA was found to be superior to CE-CCMRA in the diagnosis of significant coronary stenoses (≥50%) in patients with suspected or known CAD scheduled for conventional x-ray coronary angiography, owing to shorter scanning times and higher spatial resolution. However, CE-CCMRA and DS-CCTA have similar diagnostic accuracies.

## Introduction

### Background

Along with economic changes and modernization in developing countries, including China and India, the prevalence of atherosclerotic cardiovascular diseases is also increasing rapidly. Cardiovascular disease mortality in such countries is much higher compared to that of most developed Western countries [1]. Conventional x-ray coronary angiography is the standard technique for detecting coronary stenoses. However, x-ray angiography has limited utility as a screening tool for detecting coronary artery disease (CAD) due to its costs and invasiveness,

Over the past decade, substantial advances have been made in noninvasive cardiac imaging, including the introductions of coronary computed tomography angiography (CCTA) and cardiovascular magnetic resonance imaging (CMR), which have challenged the role of the invasive standard [2, 3]. Currently, multi-detector row spiral CCTA provides compelling images of the coronary arterial tree, and has rapidly emerged as the most promising complementary imaging test. The dual-source 64-slice CCTA (DS-CCTA) maintains the spatial resolution of the previous 64-section CT scanner with an improved temporal resolution of 83 ms [4]. In addition, CMRA represents an important modality in the assessment and management of patients with CAD. By applying 3D free-breathing coronary CMR angiography (CCMRA) to whole-heart CCMRA using a balanced steady-state free precession (bSSFP) sequence, several studies have attempted to improve the assessment of coronary artery stenoses, and to improve the image quality and provide an accurate diagnosis of clinically significant lesions [2, 5]. Recent studies have demonstrated that contrast-enhanced whole-heart coronary CCMRA at 3 T is a promising technique for imaging coronary arteries with an improved signal-to-noise ratio (SNR) [6, 7]. However, although many studies have evaluated the clinical performances of CCCT and CCMRA, to our knowledge, no study has made a direct comparison between contrast enhanced (CE)-CCMRA and DS-CCTA in the detection of significant stenoses in patients with suspected coronary artery disease.

The purpose of this current study was to prospectively compare the diagnostic performance of DS-CCTA and CE-CCMRA for the diagnosis of significant coronary stenoses (≥50%) in patients with known or suspected CAD referred for conventional coronary angiography.

### Methods

#### Study population

This prospective study was approved by the local ethics committee; all patients gave written informed consent. From June 2014 until January 2015, 74 patients with known or suspected CAD were enrolled. The patients were scheduled for conventional x-ray coronary angiography. The exclusion criteria were: absence of a sinus cardiac rhythm, orthopnea, history of previous heart surgery, presence of coronary stent, and contraindications to CMR imaging (claustrophobia, pacemaker) or CT with known allergy to iodinated contrast material, impaired renal function (creatinine > 1.4 mg/dl) and thyroid disorders.

#### Patient preparation

Patients with heart rates> 75 bpm received an oral β-blocker (metoprolol tartrate, 25–50 mg) before CE-CCMRA. In addition, 0.5 mg nitroglycerin was administered sublingually to all subjects before CE-CCMRA. A medical abdominal belt was wrapped tightly along the side of the ribs to reduce abdominal motion during deep inspiration. No β-blocker or nitroglycerin was administered to patients before DS-CCTA scans.

#### Contrast-enhanced whole-heart CMRA

CE-CCMRA was performed using a 3 T whole-body scanner (MAGNETOM Trio; Siemens Healthineers, Erlangen, Germany) with a 12-channel matrix coil (6 each for dorsal and ventral, respectively). The R-wave acquired from a three-lead wireless vectorcardiogram was used to trigger the data acquisition. The CE-CCMRA scanning procedure was as follows: after initial localization of 2-chamber and 4-chamber views and left ventricular short-axis views, a retrospective electrocardiography (ECG)-triggered cine (a fast low-angle shot (FLASH) sequence with 80 cardiac phases) was performed in the 4-chamber plane to determine the quiescent period for coronary artery imaging [8]. The acquired cine movie was visually assessed to calculate the patient-specific trigger-delay time and duration of data acquisition. For whole-heart CE-CCMRA, a prospective navigator-gated, ECG-triggered, fat-saturated, inversion recovery-prepared, segmented, three-dimensional FLASH sequence (TR 320 ms, TE 1.4 ms; flip angle 20°; matrix 256 × 256, FOV 220 × 330 mm^2^, acquired voxel size = 1.3 × 1.3 × 1.8 mm^3^ and interpolated to 0.65 × 0.65× 0.9 mm^3^) was employed. The three-dimensional k-space data were collected with a centric ordering scheme in the phase-encoding direction and a linear order scheme in the partition-encoding direction. In addition, a nonselective inversion pulse with an inversion time (TI) of 200 ms was applied before the navigator echo pulses to suppress the background tissues. In order to reduce the acquisition time, parallel imaging technique (generalized auto-calibrating partially parallel acquisitions, GRAPPA) was used in the phase-encoding direction with an acceleration factor of 2. A 0.2 mmol/kg intravenous injection of contrast agent (Gadobenate dimeglumine, Multi-Hance; Bracco Imaging SpA, Milan, Italy) was injected into an antecubital vein at a rate of 0.3 ml/s, immediately followed by 20 ml saline given at the same rate. Data acquisition began 60s after the initialization of contrast agent administration.

#### Dual-source CT coronary angiography

All patients underwent DS-CCTA (SOMATOM Definition, Siemens Healthineers). The DS-CCTA protocol included the following parameters: retrospective ECG gating; collimation, 32 × 0.6 mm; slice acquisition, 64 × 0.6 mm using the z-flying focal spot technique; gantry rotation time, 330 ms; pitch, 0.20–0.43 adapted to the heart rate; tube voltage, 100 or 120 kVp (depending on age and body mass index); and maximum tube current, 320 mAs per rotation. Spatial resolution was 0.6 × 0.6 × 0.6 mm. Temporal resolution was variable according to the heart rate of the patient.

All DS-CCTA data were acquired in deep inspiration. The volume of iodinated contrast material (Iopamidol 370 mg of iodine/ml, Bracco) was adapted to the scan time. A contrast bolus (60 to 90 ml adjusted to the individual’s body weight and scan time) was injected in an antecubital vein with a flow rate of 4 to 6 ml/s followed by a saline chaser of 40 ml at the same rate. Contrast agent application was controlled by a bolus tracking technique. The region of interest was placed into the aortic root, and image acquisition was started 7 s after the signal density level reached the predefined threshold of 100 HU.

All DS-CCTA coronary angiography datasets were reconstructed with a slice thickness of 0.75 mm and increment of 0.4 mm using medium-to-smooth convolution kernel (B26f), resulting in an inplane resolution of 0.6 to 0.7 mm and a through-plane resolution of 0.5 mm [9].

#### Conventional x-ray coronary angiography

Conventional x-ray coronary angiography served as the reference standard in this study. The diameter stenosis, as a percentage of the reference diameter, was determined in two orthogonal directions and the measurement was performed in the projection that showed the highest degree of stenosis, and was evaluated by two experienced invasive cardiologists without knowledge of the results of CE-CCMRA and DS-CCTA. All segments of the coronary artery tree with a reference diameter of 1.5 mm or greater were included in the study. A clinically significant stenosis was defined as stenosis of at least 50% of the vessel diameter.

#### CE-CCMRA and DS-CCTA data analyses

Transverse images were transferred to an off-line workstation (Siemens Healthineers). Images for each patient were analyzed in consensus by two cardiovascular radiologists with 16 and 20 years of experience. Two observers blind to the results of conventional coronary angiography evaluated the DS-CCTA and CE-CCMRA results using axial slices images, as well as multiplanar or curved reformatted reconstructions and maximum intensity projections. A 16-coronary-arterysegment model was used, based on recommendations from the American Heart Association (modified 15-segment model, with segment 16 being the intermediate branch of the left coronary artery). Image quality was assessed on a 4-point scale: 1 = poor (not assessable); 2 = fair (mild to moderate artifacts); 3 = good (minimum to mild artifacts); and 4 = excellent (minimum or no artifacts). Contrast-to-noise ratio (CNR) was evaluated in nondiseased proximal coronary segments (proximal left main (LM), mid-left anterior descending coronary artery (LAD), left circumflex coronary artery (LCX), and right coronary artery (RCA)) and computed as the difference in mean signal intensity between the vessel lumen and the adjacent tissue, divided by the standard deviation of the background noise in the proximal aorta [10]. The severity of luminal diameter reduction as being < 50% or ≥ 50% was visually assessed by two readers independently.

#### Statistical analysis

Quantitative variables were expressed as the mean value± SD, and categorical variables as percentages. CNR were compared using paired t-tests. Diagnostic performances of DS-CCTA and CE-CCMRA used x-ray coronary angiography as the standard of reference, and were presented as sensitivity, specificity, positive predictive value and negative predictive value. They were presented with the corresponding 95% confidence intervals (CIs). McNemar’s chi-square test was used to compare the diagnostic accuracy between CE-CCMRA and DS-CCTA. Inter- and intraobserver variability for image quality grading and the detection of significant coronary artery stenosis was calculated using κ statistics, and Wilcoxon test was used to compare the image quality of CE-CCMRA and DS-CCTA. Differences in accuracy between CE-CCMRA and DS-CCTA on both a patient and vessel basis were compared using McNemar’s chi-square test. Receiver operating characteristic (ROC) curves were used to assess the diagnostic efficacy and the area under the ROC curves (AUC) were compared between the two imaging modalities..A 2-tailed *P* value of 0.05 or less was considered a significant difference.

### Results

**Fig. 1:**
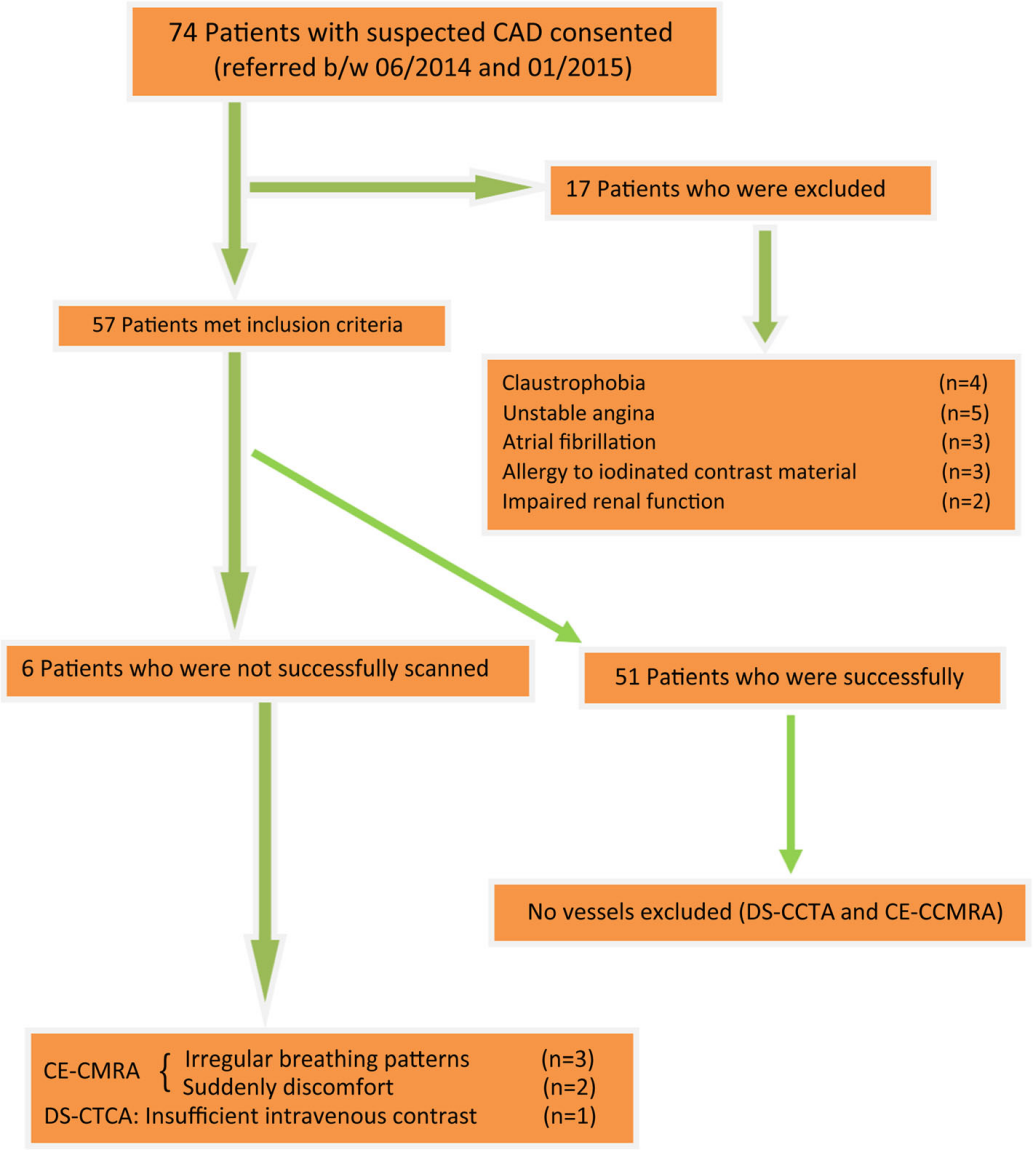
Flow chart of patient inclusion

During the study period, 74 patients were eligible for inclusion (Fig. 1). Seventeen patients were excluded for: claustrophobia(*n* = 4), unstable angina(*n* = 5), atrial fibrillation(*n* = 3), allergy to iodinated contrast material(*n* = 3), impaired renal function(*n* = 2), and seven were unsuccessfully scanned.

The final group consisted of 57 patients (40 men). Studies of six patients were discontinued because of irregular breathing patterns (n = 3) and suddenly discomfort (n = 2) during the CE-CCMRA scan, or insufficient intravenous contrast (*n* = 1) at the time of DS-CCTA. The success rate of DS-CCTA (98.2%) was higher than that of CE-CCMRA (91.2%). Thus, the final study group consisted of 51 patients (38 men; 60.2 ± 6.7 years) (Table 1). For all patients, the time interval between the application of the noninvasive tests and x-ray coronary angiography was less than 48 h. The DS-CCTA and CE-CCMRA were performed with a time interval of less than 24 h. Mean heart rates during the CE-CCMRA and DS-CCTA examinations were 65 ± 8 beats/min and 69 ± 11 beats/min, respectively. The acquisition times of CE-CCMRA and DS-CCTA, respectively, were 9.5 ± 3.1 min and 8.3 ± 1.4 s. The average navigator acceptance rate during CE-CCMRA was 36%. The CE-CCMRA was acquired during mid and late diastole (number of profiles acquired per heartbeat, segments 24–35; acquisition window, 70-102 ms; number of total shots, 225–337; number of slices, 112; nominal scan time for a 100% scan efficiency, 3:00–4:30 min; navigator accept window, ±2.5 mm).

**Table 1:** Characteristics of the Study Population

Characteristic	Value
Age (y)	60.2 ± 6.7
Sex, male/female	38/13
BMI, kg/m2	24.8 ± 2.1
Hypertension, n (%)	20 (39%)
Hypercholesterolemia, n (%)	35 (69%)
Diabetes mellitus, n (%)	19 (37%)
Current or prior cigarette smoking, n (%)	20 (39%)
Prior myocardial infarction, n (%)	16 (31%)
Family history of CAD	15 (29%)
Stenosis on x-ray coronary angiography	
1-vessel	11
2-vessel	14
3-vessel	6

Values are n (%) or mean ± SD

*CAD* coronary artery disease, *BMI* body mass index

#### Image quality of the CE-CCMRA and DS-CCTA

**Fig. 2:**
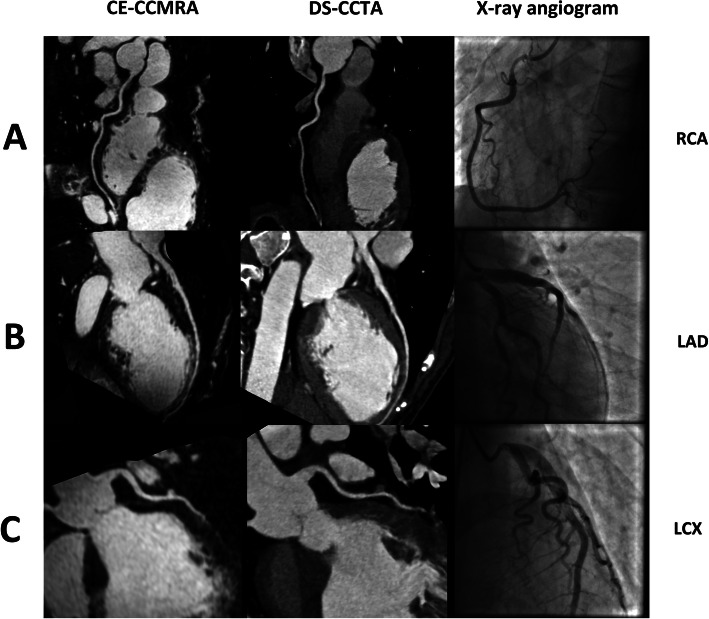
Typical examples of contrast enhanced (CE) coronary cardiovascular magnetic resonance angiography (CCMRA) (left), and dual source (DS) coronary computed tomography angiography (CCTA) (center), and corresponding x-ray angiography images (right) of the right and left coronary artery system. **a**. Normal right coronary artery (RCA). **b**. Normal left anterior descending coronary artery (LAD). **c**. Normal left circumflex coronary artery (LCX)

**Fig. 3:**
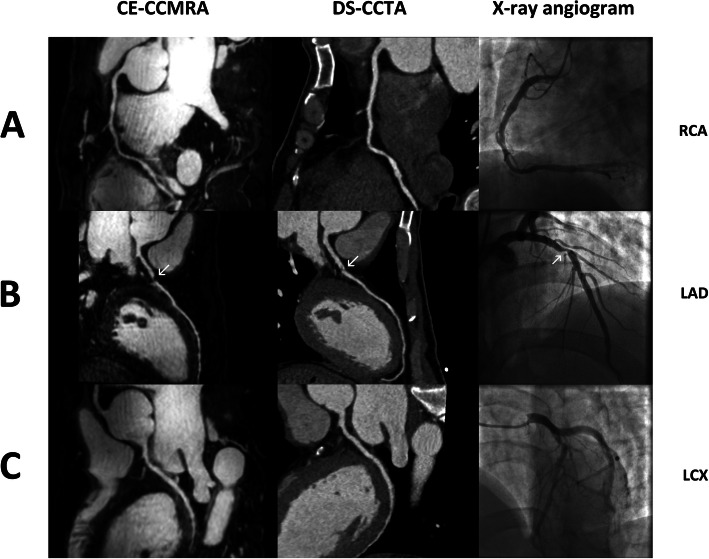
Representative example of CE-CCMRA and DS-CCTA curved planar reconstruction and corresponding x-ray angiography showing a significant stenosis in the proximal LAD (**b**), normal RCA and LCX (**a**, **c**)

The evaluation of segments by conventional x-ray coronary angiography, 3 T CE-CCMRA and DS-CCTA for 51 patients are shown in Table 2. A total of 76 of 654 coronary segments (12%) with a reference luminal diameter ≥ 1.5 mm by x-ray angiography were not available for evaluation by CE-CCMRA. However, only 35 of the 654 coronary segments (5%) with a reference luminal diameter ≥ 1.5 mm by x-ray angiography could not be visualized by DS-CCTA due to poor CNR, motion artifacts, and small diameter. For all vessels, DS-CCTA demonstrated higher image quality than CE-CCMRA. In addition, the subjectively graded image quality of segments imaged by DS-CCTA (3.5 ± 0.8) was judged to be higher than that of CE-CCMRA (3.2 ± 0.7, *p* < 0.001). The kappa values for interobserver agreement for image-quality grading were 0.86 and 0.83, respectively. Representative examples of normal coronary angiograms and coronary stenoses detected by CE-CCMRA and DS-CCTA are shown in Figs. 2 and 3, respectively.

**Table 2:** Evaluation of segments by conventional coronary angiography, contrast-enhanced coronary cardiovascular magnetic resonance angiography (CE-CCMRA) and dual-source coronary computed tomography angiography (CCTA)

Artery	No. of Segments ≥ 1.5 mm on x-ray	No. of Assessable Segments on CE-CCMRA (%)	No. of Assessable Segments on DS-CCTA (%)
**LM**	**51**	**51 (100%)**	**51 (100%)**
**LAD**			
Proximal	51	50 (98%)	51 (100%)
Middle	51	49 (96%)	51 (100%)
Distal	48	43 (90%)	46 (96%)
Diagonal branches	56	43 (77%)	50 (89%)
**LCX**			
Proximal	51	50 (98%)	51 (100%)
Distal	41	34 (83%)	37 (90%)
Marginal branches	61	39 (64%)	53 (87%)
**RCA**			
Proximal	51	50 (98%)	51 (100%)
Middle	51	49 (96%)	51 (100%)
Distal	49	46 (94%)	47 (96%)
PDA/PL	93	74 (80%)	80 (86%)
**Total**	**654**	**578 (88%)**	**619 (95%)**

*DS-CCTA* dual-source coronary computed tomography angiography, *CE-CCMRA* contrast-enhanced coronary cardiovascular magnetic resonance angiography, *LAD* left anterior descending coronary artery, *LCX* left circumflex coronary artery, *LM* left main coronary artery, *PDA/PL* posterior descending artery/posterolateral branch, *CAG* conventional coronary angiography, *RCA* right coronary artery, *CE* contrast-enhanced

#### Diagnostic performance of CE-CCMRA and DS-CCTA compared with X-ray angiography

**Fig. 4:**
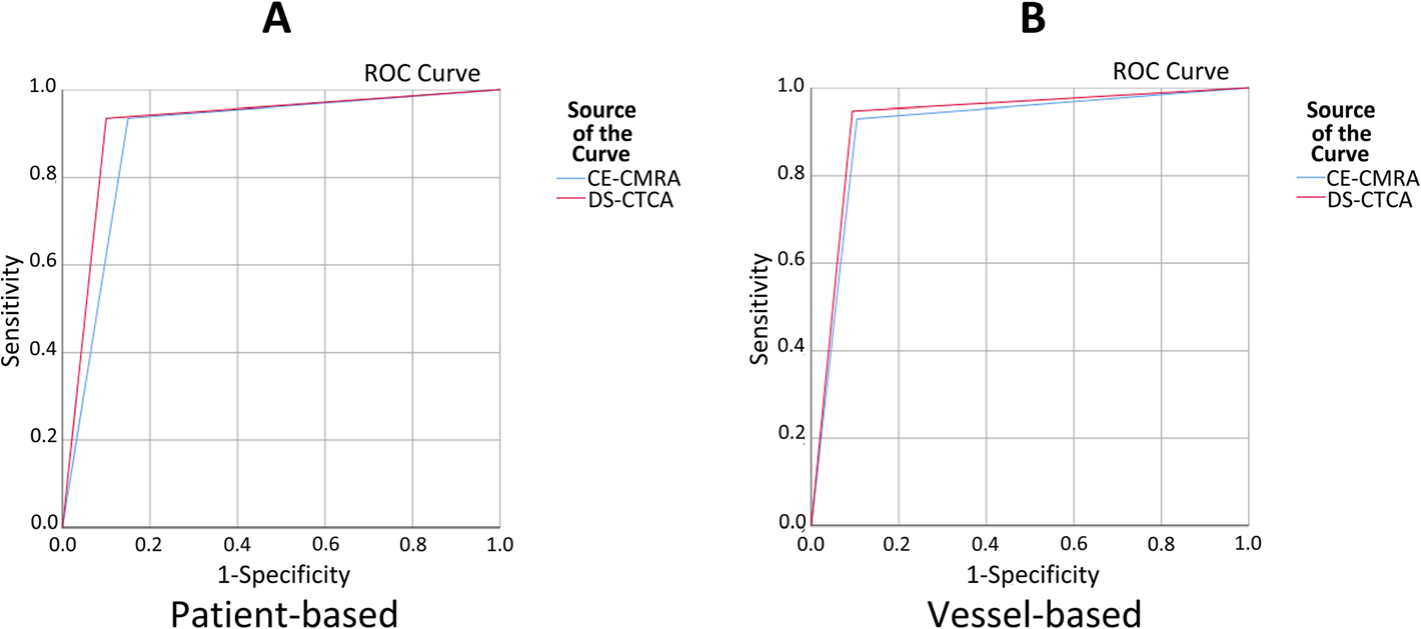
The receiver-operator characteristic (ROC) curve comparing the diagnostic performance of CE-CCMRA and DS-CCTA for detection of x-ray angiographic diameter stenosis ≥50%. (**a**) The area under the curve (AUC) was 0.89 (95% CI: 0.79 to 0.99) for CE-CCMRA and 0.92 (95% CI: 0.83 to 1.00) for DS-CCTA on the patient-based analysis. (**b**) The AUC was 0.91 (95% CI: 0.86 to 0.97) for CE-CCMRA and 0.93 (95% CI: 0.88 to 0.98) for DS-CCTA on the vessel-based analysis

The diagnostic abilities of CE-CCMRA and DS-CCTA to detect significant stenoses in a patient-, and vessel-based analysis are detailed in Table 3 and Fig. 4.

**Table 3:** Diagnostic Performance of CE-CCMRA and DS-CCTA

	CE-CCMRA	DS-CCTA	p Value
**Patient-based analysis**	***n*** **= 51**	***n*** **= 51**	
Sensitivity	93.5 (29/31) [77.2–98.9]	93.5 (29/31) [77.2–98.9]	> 0.05
Specificity	85.0 (17/20) [61.1–96.0]	90.0 (18/20) [66.9–98.2]	> 0.05
PPV	90.6 (29/32) [73.8–97.5]	93.5 (29/31) [77.2–98.9]	> 0.05
NPV	89.4 (17/19) [65.5–98.2]	90.0 (18/20) [66.9–98.2]	> 0.05
**Vessel-based analysis**	***n*** **= 153**	***n*** **= 153**	
Sensitivity	93.0 (53/57) [82.2–97.7]	94.7 (54/57) [84.5–98.6]	> 0.05
Specificity	89.6 (86/96) [81.3–94.6]	90.7 (87/96) [82.5–95.4]	> 0.05
PPV	84.1 (53/63) [72.3–91.7]	85.7 (54/63)[74.1–92.9]	> 0.05
NPV	95.6 (86/90) [88.4–98.6]	96.7 (87/90) [89.9–99.1]	> 0.05

Values are n/n (%); values in brackets are 95% confidence intervals

*NPV* negative predictive value, *PPV* positive predictive value

In the patient-based analysis, DS-CCTA correctly recognized 29 of the 31 significant stenoses detected on x-ray angiography. CE-CCMRA also correctly identified significant CAD in 29 out of 31 patients. The sensitivity (93.5%) of both tests was equally high. In contrast, CE-CCMRA had a slightly lower specificity (85%) but no significant difference in the ability to detect coronary stenosis than DS-CCTA (90%). CE-CCMRA had a AUC of 0.89 (95% CI: 0.79 to 0.99). DS-CCTA had a AUC of 0.92 (95% CI: 0.83 to 1.00). In the vessel -based analysis, the sensitivity, specificity, PPV, and NPV for detecting patients with significant CAD by DS-CCTA scan were 94.7%(54/57), 90.7%(87/96), 85.7%(54/63), and 96.7%(87/90), respectively. In addition, the diagnostic performance of CE-CCMRA on a per-vessel basis was similar, with no significant differences. The AUC was 0.91 (95% CI: 0.86 to 0.97) for CE-CCMRAand 0.93 (95% CI: 0.88 to 0.98) for DS-CCTA.

**Fig. 5:**
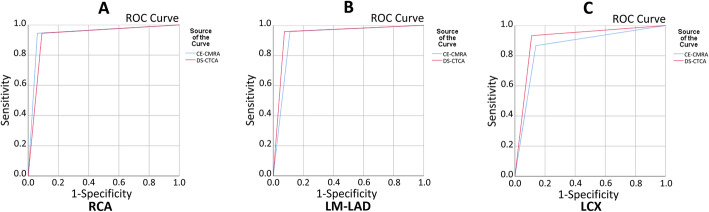
The AUC of RCA (**a**), LM-LAD (**b**), and LCX (**c**) was 0.94, 0.92, 0.86 for CE-CCMRA and 0.93, 0.94, 0.91 for DS-CCTA on the vessel-based analysis, respectively

Differences were observed for the RCA, LM-LAD, and LCX arteries (Table 4, Fig. 5). The sensitivity, specificity, positive predictive valve (PPV), negative predictive value (NPV) of the left main–LAD, RCA, LCX were similar with CE-CMRA and DS-CCTA with no significant differences. The AUC of RCA, LM-LAD, and LCX was 0.94, 0.92, 0.86 for CE-CCMRA and 0.93, 0.94, 0.91 for DS-CCTA, respectively. However, for the LCX, both CE-CCMRA and DS-CCTA had low positive predictive values.

**Table 4:** Diagnostic Accuracy of CE-CMRA and DS-CCTA for Detection of X-ray Coronary Stenosis > 50% in Different Vessels

Artery	CE-CCMRA	DS-CCTA	p Value
**RCA**			
Sensitivity	94.7 (17/18)[70.6–99.7]	94.7 (17/18)[70.6–99.7]	> 0.05
Specificity	93.9 (31/33)[78.4–98.9]	90.9 (30/33)[74.5–97.6]	> 0.05
PPV	89.5 (17/19)[65.5–98.2]	85.0 (17/20)[61.1–96.0]	> 0.05
NPV	96.9 (31/32)[82.0–99.8]	96.8 (30/31)[81.5–99.8]	> 0.05
**LM-LAD**			
Sensitivity	95.8 (23/24)[76.9–99.8]	95.8 (23/24)[76.9–99.8]	> 0.05
Specificity	88.9 (24/27) [70.0–97.1]	92.6 (25/27)[74.2–98.7]	> 0.05
PPV	88.5 (23/26)[68.7–97.0]	92.0 (23/25)[72.5–98.6]	> 0.05
NPV	96.0 (24/25)[77.7–99.8]	96.2 (25/26)[78.4–99.8]	> 0.05
**LCX**			
Sensitivity	86.7 (13/15)[58.4–97.7]	93.3 (14/15)[66.0–99.7]	> 0.05
Specificity	86.1 (31/36)[69.7–94.8]	88.9 (32/36)[73.0–96.4]	> 0.05
PPV	72.2 (13/18) [46.4–89.3]	77.8 (14/18)[51.9–92.6]	> 0.05
NPV	93.9 (31/33)[78.4–98.9]	97.0 (32/33)[82.5–99.8]	> 0.05

Values are n/n (% [95% confidence interval])

### Discussion

This present study describes on the first single-center direct comparison of CE-CCMRA and DS-CCTA to detect coronary artery stenosis in patients scheduled for conventional x-ray coronary angiography. Our study demonstrates that both DS-CCTA and CE-CCMRA can provide diagnostic accuracy in detecting significant CAD and are reliable for excluding significant CAD, without significant differences on a vessel- or a patient-based level. However, DS-CCTA provides better image quality than CE-CCMRA. We evaluated all segments being > 1.5 mm in diameter. Only 5% of the coronary segments were significantly lower than CE-CCMRA (12%). This reflected the currently better image quality of DS-CCTA. Better image quality is attributable to higher spatial resolution and CNR.

In recent years, CCTA has become widely used, and studies have demonstrated its diagnostic accuracy [11–15]. DS-CCTA represents a technical advance in CT technology and has demonstrated a high diagnostic accuracy for the assessment of significant stenoses independent of patient’s heart rate without using beta-blockers before the scan [4]. In this study, the patient-based sensitivity and specificity of DS-CCTA in the detection of significant stenoses were 93.5 and 90%, respectively, which are in close agreement with those of the previous study [4]. Moreover, our results showed that both techniques had low positive predictive values for the LCX. The main reasons for CE-CCMRA may be due to the greater distance from the receiving coils (leading to decreased SNR) and close proximity to the coronary vein both of which can confound the interpretation, and for DS-CCTA may be more severe cardiac motion due to the lack of beta blockers.

Studies during the past 20 years have shown that CMR technology allows noninvasive, radiation-free, comprehensive evaluation of CAD [2, 16, 17]. Previous research [5, 7, 18–20] reported sensitivity and specificity in the range of 70–90% using different CMR techniques. Compared with CCTA, CCMRA has several potential advantages. First, it does not expose patients to ionizing radiation. Second, the lumen of the coronary artery is not exposed to artifacts caused by coronary artery calcification [21]. Third, CMR imaging allows concurrent assessment of myocardial structure, function, myocardial viability,blood flow, and coronary arteries in a single setting.

Several studies using the direct comparison of CMR and CCTA have been carried out with different sequence types employed at different field strength, and with different generations of multislice CT [10, 22–25]. In these studies, CCMRA was performed using a non-contrast bSSFP sequence. In comparison, in our study, CE-CCMRA is employed with slow infusion of a high-relaxivity extravascular contrast agent, which improved the SNR and CNR [26–28]. In contrast to bSSFP, conventional spoiled gradient- echo sequences, such as FLASH, are less sensitive to the increased field inhomogeneities at 3 T due to the application of the spoiled gradient. Hence, we obtained CE-CCMRA at 3 T, using a navigator-gated, ECG-triggered, fat-saturated, inversion-recovery prepared segmented three-dimensional FLASH sequence. The scan time used in this study was shorter than those employed in previous studies using a conventional protocol [18]. In our study, CE-CCMRA showed a dramatically reduced acquisition time (9.5 ± 3.1 min), compared to that of a previous study (17 ± 4.7 min) [25]. The advantages of using CE-CCMRA were translated in the present study into better depiction of the coronary artery length. Yang et al. [7] demonstrated the feasibility of accurate detection of significant stenosis in a single-center study. Initial experiments have shown that CE-CCMRA provides sufficiently high sensitivity and a negative predictive value to exclude significant stenosis in patients suspected of having CAD [29]. In addition, that study [29] suggested that image quality can be further improved by sublingual nitroglycerin and abdominal banding.

In this current study, the diagnostic ability to detect significant coronary stenosis (≥50%) in CAD by visual analysis was slightly, although not significantly, higher for DS-CCTA than for CE-CCMRA. Such a difference may be caused by the higher spatial resolution and CNR of DS-CCTA, which led to a visualization of more coronary segments. Therefore, from a clinical standpoint, both DS-CCTAand CE-CCMRA provide specific advantages, considering the benefits and drawbacks of these imaging modalities, and widely increase the clinical utility of these methods.

#### Limitations

This study also has some limitations. First, the study population from the single center was relatively small. We will continue our studies with a large patient population for further subgroup analysis. Another drawback is that, after contrast administration, DS-CCTAand CE-CCMRA are not suitable for repeat examinations when the scans fail. Moreover, the time for completing CE-CCMRA procedures still remains long, and CE-CCMRA has relatively low spatial resolution. Third, the evaluation of the diagnostic performance of 3 T CE-CCMRA in this study was performed using a 12-channel cardiac coil. Recently, a 32-channel cardiac coil has become commercially available, which would reduce the imaging time of cardiac imaging [30], and could offer even higher diagnostic accuracy [31]. Finally, a limitation of this approach is that it may be difficult to combine a high dose CE-CCMRA scan with a rest and stress perfusion CMR protocol which would require additional gadolinium.

### Conclusions

In conclusion, DS-CCTA is superior to 3 T CE-CCMRA in the diagnosis of significant CAD in patients with suspected or known CAD scheduled for conventional x-ray coronary angiography. DS-CCTA has shorter scanning times and higher spatial resolution. However, 3 T CE-CCMRA and DS-CCTA have similar diagnostic accuracies.

## Data Availability

All data generated or analyzed during this study are included in this published article.

## Abbreviations

AUC: Area under the curve
bSSFP Balanced steady state free precession CAD: Coronary artery disease
CCMRA: Contrast-enhanced coronary cardiovascular magnetic resonance angiography
CE: Contrast enhanced
CCTA: Coronary computed tomography angiography
CMR: Cardiovascular magnetic resonance
CNR: contrast-to-noise ratio
DS Dual source ECG: Electrocardiogram
FLASH: Fast low-angle shot
LAD: Left anterior descending coronary artery
LCX: Left circumflex coronary artery
LM: Left main coronary artery
NPV: Negative predictive value
PPV: Positive predictive value
RCA: Right coronary artery
ROC: Receiver operating characteristics
SNR: Signal-to-noise ratio TI Inversion time
